# Generating Excess Protons in Microsolvated Acid Clusters under Ambient Conditions: An Issue of Configurational Entropy versus Internal Energy

**DOI:** 10.1002/chem.202000864

**Published:** 2020-08-20

**Authors:** Ricardo Pérez de Tudela, Dominik Marx

**Affiliations:** ^1^ Lehrstuhl für Theoretische Chemie Ruhr-Universität Bochum 44780 Bochum Germany

**Keywords:** ab initio calculations, acid dissociation, entropy, solvation, thermodynamic integration

## Abstract

Acid dissociation, and thus liberation of excess protons in small water droplets, impacts on diverse fields such as interstellar, atmospheric or environmental chemistry. At cryogenic temperatures below 1 K, it is now well established that as few as four water molecules suffice to dissociate the generic strong acid HCl, yet temperature‐driven recombination sets in simply upon heating that cluster. Here, the fundamental question is posed of how many more water molecules are required to stabilize a hydrated excess proton at room temperature. Ab initio path integral simulations disclose that not five, but six water molecules are needed at 300 K to allow for HCl dissociation independently from nuclear quantum effects. In order to provide the molecular underpinnings of these observations, the classical and quantum free energy profiles were decomposed along the dissociation coordinate in terms of the corresponding internal energy and entropy profiles. What decides in the end about acid dissociation, and thus ion pair formation, in a specific microsolvated water cluster at room temperature is found to be a fierce competition between classical configurational entropy and internal energy, where the former stabilizes the undissociated state whereas the latter favors dissociation. It is expected that these are generic findings with broad implications on acid–base chemistry depending on temperature in small water assemblies.

## Graphical abstract

**Temperature dependent**: Extensive ab initio path integral simulations disclose that the number of water molecules required to allow for acid dissociation, and thus for the generation of hydrated excess protons, in restricted aqueous environments such as finite HCl(H_2_O)_*n*_ clusters, is temperature dependent.

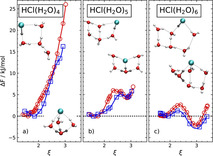

One of the most fundamental reactions in chemistry with widespread implications is the generation of excess protons as a result of acid dissociation in aqueous environments. Since decades, special attention has been paid to such and similar reactions subject to a restricted availability of water molecules, such as water droplets,^1^, ^2^, ^3^, ^4^, ^5^ ice nanoparticles^6^, ^7^, ^8^, ^9^, ^10^, ^11^, ^12^, ^13^, ^14^ and small HCl/water clusters at low temperatures.^15^, ^16^, ^17^, ^18^, ^19^, ^20^, ^21^, ^22^, ^23^, ^24^, ^25^, ^26^, ^27^ Much of the motivation comes from the importance of chlorine compounds, HCl in the first place, and its reaction products with water, notably excess protons, in atmospheric and interstellar processes at low and ultra‐low temperatures. In case of HCl/water clusters, it is only after many years of research and controversies^15^, ^16^, ^17^, ^18^, ^19^, ^20^, ^21^, ^22^, ^23^, ^24^, ^25^, ^26^, ^27^ that it is now firmly established that as few as four water molecules are required to dissociate HCl at ultra‐low temperatures.^19^, ^27^

In stark contrast to all this previous research, we focus here on the temperature sensitivity of this reaction, having in mind acid‐catalyzed processes at the typical environmental and physiological conditions of about 300 K. Indeed, there is some evidence in the literature for the stabilization of molecular HCl^17^, ^24^, ^25^, ^26^ and recombination of salt ions^28^ in small water clusters simply by raising the temperature. However, whereas at room temperature, acid dissociation, excess protons and proton transfer have been extensively investigated in bulk water,^29^, ^30^, ^31^ the same cannot be said when it comes to limiting the amount of water to only a few molecules. As will be disclosed, moving away from the ground state behavior relevant to cryochemistry by considering thermal activation phenomena contributes new facets to acid dissociation and recombination in the microsolvation limit.

Given this background, we now pose the apparently simple but fundamental question of how many water molecules are required at room temperature to generate excess protons in small water clusters by dissociating HCl therein. To this end, we perform extensive ab initio path integral and ab initio molecular dynamics simulations combined with ab initio thermodynamic integration of HCl(H_2_O)_*n*_ clusters with *n*=4, 5 and 6 at 300 K (see Ref. 32 for methodological background) making use of the CP2K program package.^33^ All electrons were taken into account explicitly using the BLYP density functional along with the aug‐cc‐pVTZ basis set as provided in CP2K; we refer to previous work on HCl/water clusters for validation of this approach.^26^, ^34^, ^35^, ^36^

Computational details, references as well as supporting analyses are compiled in the Supporting Information. Importantly, we carefully and specifically assessed previously the performance of the BLYP functional for the present task based on MP2 and Coupled Cluster benchmark calculations, including several cluster sizes, isomers and dissociation states, as reported in supporting Table II and Table S1 of refs. 26 and 27.

The classical and quantum free energy profiles computed at room temperature for HCl(H_2_O)_*n*_ clusters, see Figure 1, unveil a distinctly different HCl dissociation behavior upon stepwise addition of water molecules from *n*=4 to 5 to 6. The dimensionless dissociation coordinate *ξ*, which allows us to distinguish the undissociated (U) and dissociated (D) states of a given cluster size, is given by the coordination number of the Cl site with respect to all O sites (i.e., CN_Cl–O_ as defined in the Supporting Information). The insets depict the respective U and D structures corresponding roughly to *ξ*≈1.7 and ≈3.0, respectively, which is rather independent from the cluster size *n*. Our previous finding^26^ that the HCl molecule in the *n*=4 cluster is undissociated at 300 K is confirmed here based on improved statistics (but recall that the dissociated state of HCl(H_2_O)_4_ has been shown to be preferred below roughly 200 K). The obvious conclusion is that the smallest droplet of dissociated acid at ambient conditions must be larger than only four water molecules, which therefore holds only true at sufficiently cold conditions.^19^, ^27^

**Figure 1:**
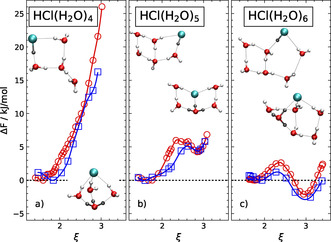
Classical (red circles) and quantum (blue squares) Helmholtz free energy profiles, Δ*F* (*ξ*), as a function of the dimensionless dissociation coordinate *ξ* (see text) which connects the undissociated (U) species (at *ξ*≈1.7) to the dissociated (D) species (at *ξ*≈3.0) for the HCl(H_2_O)_4_, HCl(H_2_O)_5_ and HCl(H_2_O)_6_ clusters (from left to right) at 300 K; the respective optimized equilibrium structures of the U/D species are depicted as upper/ lower insets in each panel.

Interestingly, whereas the *n*=4 cluster does not feature any stability of the dissociated state at all, since the free energy is monotonically increasing along the dissociation coordinate *ξ* when leaving the undissociated minimum at *ξ*≈1.7 according to Figure 1 a, the dissociated state of the *n*=5 cluster is found to correspond to a local free energy minimum at *ξ*≈3.0, see Figure 1 b. This heralds a metastable hydrated ion pair in the case of HCl(H_2_O)_5_ at room temperature. Based on these data, we conclude that five water molecules are not yet sufficient to generate an excess proton by allowing HCl to dissociate. Upon adding one more water molecule, thus reaching HCl(H_2_O)_6_, the situation is found to be qualitatively different from both previous scenarios since now, for *n*=6, the dissociated state turns out to be lower in free energy in Figure 1 c.

Having analyzed the free energies presented in Figure 1, which have been obtained from explicit ab initio simulations at 300 K, we mention in passing that standard quantum‐chemical thermochemistry calculations of free energy differences based on optimized structures (as recently performed in Ref. 25 to study HCl/water clusters) erroneously predict the dissociated *n*=5 cluster to be globally stable at 300 K. This claim is based on data generated by us using the BLYP/aug‐cc‐pVTZ approach as in our explicit ab initio simulations in conjunction with consistently optimized dissociated and undissociated cluster structures, and after having confirmed for *n*=4 that our electronic structure method closely reproduces the potential energy surface as provided by the more sophisticated dispersion‐corrected double‐hybrid B2PLYP‐D3/def2‐TZVP approach, which has been employed in Ref. 25 in the present context. In the other two cases, *n*=4 and 6, our static thermochemistry calculations of free energy differences at 300 K were in qualitative accord with the reported results of the explicit ab initio simulations at 300 K. This methodological aside makes clear that anharmonicities and configurational entropies need to be properly accounted for at finite temperatures when assessing the relative thermodynamic stabilities of dissociated versus undissociated HCl/water clusters as a function of temperature.

Finally, we address the question of whether this scenario discussed so far based on the classical free energies compiled in Figure 1 depends on nuclear quantum effects given that proton transfer processes and relative stabilities of different structures of (protonated) water clusters might be prone to zero‐point motion effects and/or tunneling phenomena depending on the case. Qualitative inspection of the data compiled in Figure 1 demonstrates that nuclear quantum effects contribute only marginally to both the relative stabilities of dissociated versus undissociated species and the interconversion free energy barriers.

Is there a way to understand the observed behavior, that is, why does the dissociated HCl molecule hosted within the *n*=4 cluster recombine upon increasing the temperature to 300 K? And why is *n*=6 the smallest cluster size that allows for stabilization of an ion pair and thus HCl dissociation at ambient conditions? In an effort to tackle these questions, we significantly extended our previous simulations of the HCl(H_2_O)_4_ cluster^26^ in order to generate a statistical data quality that allows us to separate the Helmholtz free energy profiles Δ*F*(*ξ*) into the corresponding difference of internal energies Δ*U*(*ξ*) and entropies Δ*S*(*ξ*) along the dissociation coordinate *ξ* for the different temperatures considered, as depicted in Figure 2; it must be noted that −*T*Δ*S*(*ξ*) is depicted to provide an energy scale corresponding to that of Δ*F*(*ξ*) and Δ*U*(*ξ*).

**Figure 2:**
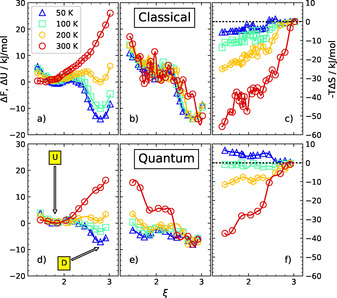
Classical (upper panels) and quantum (lower panels) Helmholtz free energy profiles (left panels and left scale: Δ*F*), internal energy profiles (middle panels and left scale: Δ*U*), and entropy profiles (right panels and right scale: −*T*Δ*S*) of the HCl(H_2_O)_4_ cluster as a function of the dimensionless dissociation coordinate *ξ* (see text) at 50 K (blue triangles), 100 K (green squares), 200 K (yellow diamonds) and 300 K (red circles); the positions of the free energy minima corresponding to undissociated (U) and dissociated (D) species are highlighted in panel d by arrows.

In the limit of classical thermal fluctuations, the internal energy profile Δ*U*(*ξ*) of HCl(H_2_O)_4_ is found to be essentially temperature‐independent with the global minimum corresponding to the dissociated state. This immediately implies that this D state—and therefore acid dissociation—is energetically (!) preferred even at room temperature, see Figure 2 b. However, the associated entropy change Δ*S*(*ξ*) is always negative upon dissociation (since *T*Δ*S*(*ξ*) increases from U to D along the dissociation coordinate according to Figure 2 c), which means that the undissociated state has a higher configurational entropy compared to the dissociated one. High Δ*S* values at a given temperature imply entropic stabilization at the level of free energy differences since Δ*F*=Δ*U*−*T*Δ*S*. The resulting purely entropic stabilization of the U state of the HCl(H_2_O)_4_ cluster gets significantly more pronounced the higher the temperature is, until finally, at 300 K, entropy wins over the (roughly constant internal) energy differences between the U and D states. In other words: Although dissociation is energetically preferred at all (!) temperatures, it becomes increasingly penalized due to the increasing entropy gain that favors the undissociated cluster species, which ultimately overrides energy when reaching 300 K (whereas there is close to perfect energy/entropy compensation observed at 200 K, leading to essentially degenerate free energies of the U and D *n*=4 clusters, cf. yellow diamonds in Figure 2 a).

The picture just described, which exclusively considers thermal activation in view of considering the nuclei to behave like classical point particles, changes remarkably when considering nuclear quantum effects in addition to thermal fluctuations (although the net trends at the level of the resulting free energies remains qualitatively unchanged according to Figure 2 d). Purely energetically, that is, at the level of the quantum internal energy differences Δ*U*(*ξ*), the dissociated state is found to be only marginally more stable than the undissociated species at the lowest temperature considered, as evidenced by the rather flat blue line in Figure 2 e. Yet, the quantum free energy of D is much lower than that of the U state according to Figure 2 d. This is only possible since the quantum entropy change is negative (!) upon dissociation (as nicely seen by the blue triangles in Figure 2 f which are always above the −*T*Δ*S*(*ξ*)=0 kcal mol^−1^ dotted horizontal line), which is exactly opposite to the entropy effect that is induced by the purely thermal fluctuations of classical nuclei (meaning that the blue triangles in Figure 2 c are always below the line of zero entropy change along dissociation). In other words: Quantum fluctuations of the nuclei stabilize the dissociated state of the HCl(H_2_O)_4_ cluster at low temperatures (whereas purely thermal fluctuation stabilize the undissociated state instead); we note in passing that quantum entropy at *T*=0 K is exclusively due to the zero‐point motion of the nuclei (i.e., zero‐point vibrations) whereas the situation is more complex at any finite temperature, *T*>0 K, such as considered here throughout. Interestingly, the quantum entropies of the U and D states are essentially identical at 100 K (since the green squares in Figure 2 f remain close to the zero‐entropy change line along dissociation), thus leaving the internal energy profile Δ*U*(*ξ*) largely unaffected at that temperature, which therefore fully determines the corresponding free energy change Δ*F*(*ξ*) upon dissociation in Figure 2 d. At room temperature, as expected, nuclear quantum effects become much less important compared to the increasingly large thermal fluctuations such that the quantum internal energy and entropy profiles (i.e., red circles in panels e and f in Figure 2) nicely approach their classical limits (i.e., red circles in panels b and c, respectively).

Based on this detailed analysis, we can provide a surprising answer to the first question: the classical configurational entropy is exclusively responsible for stabilizing the undissociated HCl(H_2_O)_4_ cluster at room temperature, while the quantum entropy due to nuclear quantum fluctuations greatly stabilizes the dissociated HCl(H_2_O)_3_(H_3_O)^+^ state at low temperatures. This entirely entropic mechanism explains fundamentally the enormous stability of the dissociated *n*=4 cluster at low temperatures as well as the previously observed proton transfer from water back to chloride when reaching ambient conditions.

In order to finally address the high‐temperature behavior when moving from *n*=4 to the two larger clusters, we also decompose their free energy profiles at 300 K into internal energy and entropy contributions. The resulting classical and quantum entropy profiles of the HCl(H_2_O)_4_, HCl(H_2_O)_5_ and HCl(H_2_O)_6_ clusters (left to right) along the dissociation coordinate are depicted in Figure 3; note that +*T*Δ*S*(*ξ*) is now plotted for convenience. Although the data are noisy since two large quantities (internal energy and negative entropy) add up to a much smaller sum (free energy) from which they are determined computationally, the observed general trend of the resulting entropy changes along the *ξ* coordinate is qualitatively robust. As can be seen from Figure 3, the configurational entropy is always much larger when HCl is undissociated (where *ξ*≈1.7) compared to the dissociated species (found around *ξ*≈3.0), meaning *T*Δ*S*(*ξ*≈1.7)≫*T*Δ*S*(*ξ*≈3.0) at constant T=300 K. This implies that the undissociated acid is consistently entropically stabilized at ambient temperature irrespective of the cluster size. Moreover, the classical (red circles) and quantum (blue squares) entropy changes show a very similar trend in all three cases, especially when HCl is microsolvated using *n*=5 and 6 water molecules, where both curves can hardly be distinguished within the statistical noise. Thus, nuclear quantum effects and the resulting quantum fluctuations can be safely neglected when analyzing Δ*S* at ambient temperature not only for *n*=4, as already shown as a function of increasing temperature in Figure 2, but also for *n*=5 and 6 at 300 K based on the data in Figure 3. Moreover, both the classical and quantum internal energies Δ*U* at 300 K are found to be lower in the dissociated state for all cluster sizes akin to what is shown in Figure 2 for *n*=4 in the middle panels. This implies that (internal) energies consistently favor HCl dissociation and thus ion pair formation at ambient temperature, in stark contrast to entropy.

**Figure 3:**
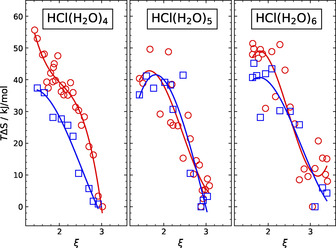
Classical (red circles) and quantum (blue squares) entropy profiles, +*T* Δ*S*(*ξ*), as a function of the dimensionless dissociation coordinate *ξ* (see text) which connects the undissociated (U) species (at *ξ*≈1.7) to the dissociated (D) species (at *ξ*≈3.0) for the HCl(H_2_O)_4_, HCl(H_2_O)_5_ and HCl(H_2_O)_6_ clusters (from left to right) at 300 K. It must be noted that the lines exclusively serve as a guide to the eye and thus carry no other information.

Evidently, entropy and internal energy compete at ambient temperature largely independently from nuclear quantum effects since they favor different species, as decided in the end by the resulting global free energy minimum, namely undissociated and dissociated HCl/water clusters, respectively. Considering now numbers, the battle between internal energy and configurational entropy at ambient temperature is won by entropy in case of the small *n*=4 cluster, thus rendering the undissociated species stable at 300 K. With *n*=6 water molecules, HCl is allowed to dissociate since the corresponding entropy loss is overcompensated by the gain in internal energy upon formation of the microsolvated ion pair. Borderline is the HCl(H_2_O)_5_ cluster since its global free energy minimum corresponds to the undissociated state, yet there is already a local minimum present, corresponding to the dissociated state, which is only a few kcal mol^−1^ higher in free energy. In that sense, the *n*=5 species with its “nascent ion pair state” (in the sense of thermodynamic metastability) in a way announces the size‐dependent transition from molecular to dissociated HCl in microsolvated water clusters at ambient temperatures.

In conclusion, our extensive ab initio path integral simulations disclose that the number of water molecules required to allow for acid dissociation, and thus for the generation of hydrated excess protons in restricted aqueous environments such as finite HCl(H_2_O)_*n*_ clusters, is temperature dependent. For a prototype acid, HCl, we show that at least *n*=6 water molecules are required at room temperature, whereas only *n*=4 water molecules suffice to induce dissociation at low temperatures. Decomposition of the classical and quantum Helmholtz free energy profiles along the HCl dissociation coordinate in terms of their internal energy and entropy components discloses that the classical configurational entropy is exclusively responsible for stabilizing the undissociated HCl(H_2_O)_4_ molecular species at room temperature, while the quantum entropy due to nuclear quantum fluctuations greatly stabilizes the dissociated HCl(H_2_O)_3_(H_3_O)^+^ ion pair at low temperatures. This entirely entropic mechanism explains fundamentally the enormous stability of the dissociated *n*=4 cluster at low temperatures as well as recombination of HCl upon increasing the temperature to 300 K. Next, it is found that the undissociated species are entropically stabilized at room temperature irrespective of the cluster size and independently from additional nuclear quantum fluctuations. The internal energy, in stark contrast, favors in all cases the dissociated state and thus ion pair formation at 300 K, which is also not altered when including nuclear quantum effects. In the end, it is the salient competition of classical conformational entropy and internal energy that decides at room temperature about the global free energy minimum of microsolvated HCl depending on cluster size: Entropy wins at 300 K in the case of *n*=4, whereas internal energy wins for *n*=6. The cluster size in between, *n*=5, is found to represent a “nascent ion pair state”, since the undissociated species is thermodynamically stable, but the dissociated ion pair is a higher‐lying local free energy minimum at room temperature corresponding to a metastable state. There is no reason not to expect similar fundamental mechanisms to be operational in many other cases as well, which is why we believe that the reported phenomenon is of general nature. An immediate consequence is that the efficiency to generate excess protons in the presence of only a limited number of water molecules is distinctly different at cryochemical conditions, used for example in high‐resolution spectroscopy, compared to that at room temperature, which is relevant to environmental and physiological processes.

## Conflict of interest

The authors declare no conflict of interest.

## Supporting information

As a service to our authors and readers, this journal provides supporting information supplied by the authors. Such materials are peer reviewed and may be re‐organized for online delivery, but are not copy‐edited or typeset. Technical support issues arising from supporting information (other than missing files) should be addressed to the authors.

Supplementary
