# Supplementary material for: Generating Excess Protons in Microsolvated Acid Clusters under Ambient Conditions: An Issue of Configurational Entropy versus Internal Energy
Source: Chemistry. 2020 Aug 20;26(52):11955–9. doi: 10.1002/chem.202000864 (PMC7540491; doi:10.1002/chem.202000864)
Supplement: Supplementary file 1 — Supplementary [file CHEM-26-11955-s001.pdf]

Chemistry–A European Journal

Supporting Information

Generating Excess Protons in Microsolvated Acid Clusters under Ambient Conditions: An Issue of Configurational Entropy versus Internal Energy

Ricardo Pérez de Tudela* and Dominik Marx[a]

COMPUTATIONAL DETAILS

All structure optimization, ab initio thermodynamic integration as well as the ab initio path integral and ab initio molecular dynamics simulations [1] were performed using the `CP2K` program package [2] together with the BLYP density functional and the aug-cc-pVTZ basis set. The suitability of the BLYP functional for the study of HCl/water clusters has been thoroughly assessed in previous works [3–7], where we refer in particular to the detailed benchmarks using essentially converged coupled cluster calculations as reported in the Supplementary Materials of Ref. [7].

The ab initio thermodynamic integration calculations [1] with classical and quantum nuclei were performed for $HCl(H_2O)_4$, $HCl(H_2O)_5$ and $HCl(H_2O)_6$ at 300 K (and compared with the 300 K results from our previous work [8] of only $HCl(H_2O)_4$ obtained with less statistics). The choice of the starting configurations necessary to carry out thermodynamic integration as well as the choice of the reaction coordinate, i.e. the order parameter that enforces HCl dissociation, are explained in detail in the following sections. The length of the constrained trajectories varied in the range of around 50 to 100 ps for the classical simulations and 40 to 60 ps for the quantum ones as determined by ensuring for each value of the order parameter the convergence of the averaged constraining force. For the centroid–constrained ab initio path integral simulations as required for quantum thermodynamic integration, eight Trotter replica or beads were sufficient at 300 K.

INITIAL CONFIGURATIONS FOR THERMODYNAMIC INTEGRATION

In order to calculate the free energy profiles between dissociated (D) and undissociated (U) species of $HCl(H_2O)_n$ clusters, with $n = 4, 5, 6$, the thermodynamic integration technique has been employed. Intermediate configurations between D and U along the dissociation path are needed as initial seeds to efficiently launch the thermodynamic integration procedure, which have been generated as follows.

For $HCl(H_2O)_4$, the free energy profile between the undissociated dissociated species has been computed in our previous work [8] to which we refer the reader for the details.

For $HCl(H_2O)_5$, the most relevant structures were taken from Ref. [9] but reoptimized using our electronic structure approach as described in the previous section, yielding those configurations that are depicted in Fig. 1. For HCl with five water molecules, the minimum of the potential energy surface corresponds to a book–shaped structure in which the HCl molecule is dissociated, the chloride and the hydronium being located along the book gutter. Both ions are directly bound by a hydrogen bond, thus leading to a so–called contact ion pair structure D_5. The undissociated minimum, called here U_5, retains the same overall structure but in this case the HCl molecule resides on one of the sides of the book. The pathway that converts the U_5 into the D_5 species can be visualized as the "closing" of the book: Once both ends meet, a water molecule donates an extra hydrogen bond to the HCl, which immediately dissociates and leaves the Cl^- accepting three hydrogen bonds. Then the chloride prefers to stay in the middle of the cluster, "pushing" the gutter towards the side. Selected structures along this pathway were employed as seeds for the ab initio thermodynamic integration calculations.

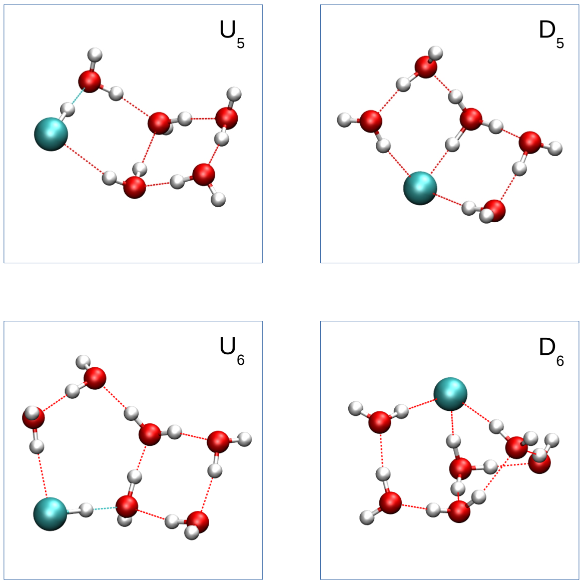

Figure 1. Relevant undissociated (U) and dissociated (D) $HCl(H_2O)_5$ and $HCl(H_2O)_6$ structures as described and discussed in the text.

For $HCl(H_2O)_6$, a series of about 20 ps long ab initio molecular dynamics trajectories was generated at 400 K, using different U and D starting structures that had been obtained by structure optimizations using a manifold of structures generated from optimized $HCl(H_2O)_5$ clusters where an additional water molecule has been randomly attached. From a representative trajectory in which several U–D interconversions took place, snapshots of the transition between U and D were used as starting configurations for the thermodynamic integration calculations. In particular, the representative undissociated and dissociated species are shown in Fig. 1 as U_6 and D_6, respectively, after reoptimization. The initial undissociated conformer U_6 can be visualized as a planar pentagonal arrangement of four water molecules and the HCl molecule

attached to two other waters that are bound among themselves, whereas the dissociated species is again a contact ion pair. Also the pathway that connects U and D in this case shares some similarities with that of the $HCl(H_2O)_5$ dissociation process. The two outer water molecules start to approach the pentagonal ring in the same fashion as in the afore–described "book closing" in case of $n = 5$. As soon as one of the outermost waters donates a hydrogen bond to HCl, the dissociation takes place and a total rearrangement of the molecules occur, along with proton transfer.

COORDINATION NUMBER AS THE ORDER PARAMETER

In some previous work on the $HCl(H_2O)_4$ cluster [10], the free energy surface was computed using the ab initio metadynamics technique [11], making use of two so–called collective variables, namely the coordination number (CN) between the chloride with respect to all oxygens and the torsional angle formed by the four oxygen atoms. On the generated surface, undissociated species fall in regions with a CN close to two, while dissociated species are cleanly located in separate areas with CN close to three. The definition of the CN variable is as follows

$$\mathrm{CN}_{\mathrm{Cl-O}} = \frac{1}{N_{\mathrm{O}}} \sum_{i=1}^{N_{\mathrm{O}}} \frac{1 - \left(\frac{R_i}{R_0}\right)^l}{1 - \left(\frac{R_i}{R_0}\right)^m} \ , \tag{1}$$

where N_{O} is the number of oxygen atoms in the cluster, R_i is the distance of the oxygen i to the chloride atom; R_0, l and m are fixed parameter with values of 3.7Å, 6 and 12, respectively. The order parameter denoted as ξ in the main text was chosen to be this coordination number, $\mathrm{CN}_{\mathrm{Cl-O}}$, to consistently describe the HCl dissociation process in all $HCl(H_2O)_n$ clusters.

In order to ensure the suitability of $\mathrm{CN}_{\mathrm{Cl-O}}$ to correctly characterize the dissociation processes also for the two larger clusters, we have computed the fraction of un/dissociated species contributing to the thermodynamic integration sampling at each contraint value, see Fig. 2. The data in the top and bottom panels demonstrate that for five and six water molecules, respectively, coordination number values below ≈ 2 and above ≈ 3 indeed correspond to undissociated and dissociated such clusters. Values intermediate between two and three correspond to a superposition of the two species as expected for the transition regime between the undissociated to dissociated minima along the free energy profile shown in the main text.

With this analysis, it can be concluded that the coordination number $\mathrm{CN}_{\mathrm{Cl-O}}$ can be used as an order parameter ξ to describe HCl dissociation in water clusters with at least up to six water molecules.

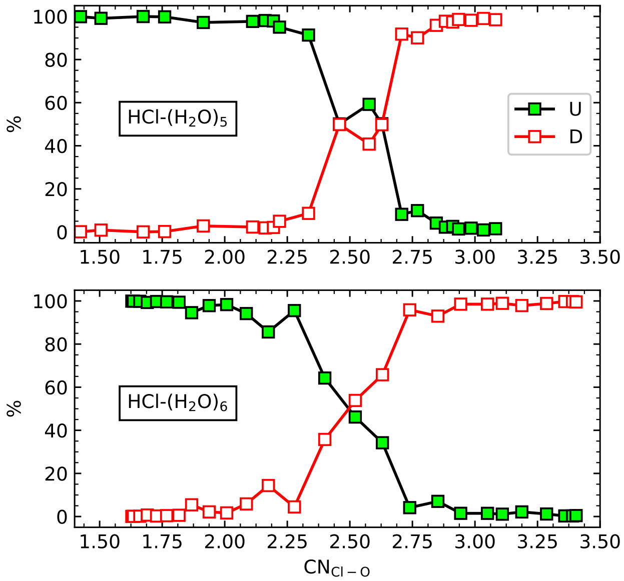

Figure 2. Fraction (in percent) of the constrained trajectories using classical nuclei in which the HCl molecule is undissociated (U, green full symbols) and dissociated (D, red open symbols) for $HCl(H_2O)_5$ (top panel) and $HCl(H_2O)_6$ (bottom panel) both at 300 K as a function of the coordination number, $\mathrm{CN}_{\mathrm{Cl-O}}$.

[1] D. Marx and J. Hutter, *Ab Initio Molecular Dynamics: Basic Theory and Advanced Methods* (Cambridge University Press, Cambridge, 2009).
[2] The CP2K Developers Group, http://www.cp2k.org.
[3] M. Masia, H. Forbert, and D. Marx, "Connecting structure to infrared spectra of molecular and autodissociated HCl–water aggregates," J. Phys. Chem. A **111**, 12181–12191 (2007).
[4] Ł. Walewski, H. Forbert, and D. Marx, "Quantum induced bond centering in microsolvated HCl: Solvent separated versus contact ion pairs," J. Phys. Chem. Lett. **2**, 3069–3074 (2011).
[5] Ł. Walewski, H. Forbert, and D. Marx, "Revealing the subtle interplay of thermal and quantum fluctuation effects on contact ion pairing in microsolvated HCl," ChemPhysChem **14**, 817–826 (2013).
[6] Ł. Walewski, H. Forbert, and D. Marx, "Solvation of molecules in superfluid helium enhances the interaction induced localization effect," J. Chem. Phys. **140**, 144305 (2014).
[7] D. Mani, R. Pérez de Tudela, R. Schwan, N. Pal, S. Körning, H. Forbert, B. Redlich, A. F. G. van der Meer, G. Schwaab, D. Marx, and M. Havenith, "Acid solvation versus dissociation at "stardust conditions": Reaction sequence matters," Sci. Adv. **5**, eaav8179 (2019).
[8] R. Pérez de Tudela and D. Marx, "Acid dissociation in HCl-water clusters is temperature dependent and cannot

be detected based on dipole moments," Phys. Rev. Lett. **119**, 223001 (2017).

[9] A. Vargas-Caamal, J. L. Cabellos, F. Ortiz-Chi, H. S. Rzepa, A. Restrepo, and G. Merino, "How many water molecules does it take to dissociate HCl?" Chem. Eur. J. **22**, 2812–2818 (2016).

[10] H. Forbert, M. Masia, A. Kaczmarek-Kedziera, N. N. Nair, and D. Marx, "Aggregation-induced chemical reactions: Acid dissociation in growing water clusters," J. Am. Chem. Soc. **133**, 4062–4072 (2011).

[11] A. Laio and M. Parrinello, "Escaping free-energy minima," Proc. Natl. Acad. Sci. **99**, 12562–12566 (2002).
